# Goodness-of-fit test for meta-analysis

**DOI:** 10.1038/srep16983

**Published:** 2015-11-23

**Authors:** Zhongxue Chen, Guoyi Zhang, Jing Li

**Affiliations:** 1 Department of Epidemiology and Biostatistics, School of Public Health, Indiana University Bloomington, 1025 E. 7th street, Bloomington, IN 47405, USA.; 2 Department of Mathematics and Statistics, University of New Mexico, Albuquerque, NM 87131, USA

## Abstract

Meta-analysis is a very useful tool to combine information from different sources. Fixed effect and random effect models are widely used in meta-analysis. Despite their popularity, they may give us misleading results if the models don’t fit the data but are blindly used. Therefore, like any statistical analysis, checking the model fitting is an important step. However, in practice, the goodness-of-fit in meta-analysis is rarely discussed. In this paper, we propose some tests to check the goodness-of-fit for the fixed and random effect models with assumption of normal distributions in meta-analysis. Through simulation study, we show that the proposed tests control type I error rate very well. To demonstrate the usefulness of the proposed tests, we also apply them to some real data sets. Our study shows that the proposed tests are useful tools in checking the goodness-of-fit of the normal models used in meta-analysis.

Due to technological advancement, the speed of generating large data is increasing. The tremendous amount of data provide us opportunities to answer many scientific questions. On the other hand, however, we are facing the challenge: how to extract useful information from different but related studies. Meta-analysis has been shown to be a very useful tool to combine information; it has being intensively used in data analysis^1^. For example, when we used “((meta-analy* or metaanaly* or metanaly* or pooled analy* or consorti*) or meta-analysis)” to retrieve researches on meta-analysis from the PubMed database (http://www.ncbi.nlm.nih.gov/pubmed), it returned 65,881 papers published within the most recent five years (as of February 8, 2015). This demonstrates that meta-analysis is a popular and useful statistical tool in data analysis.

Fixed effect (FE) model and random effect (RE) model are the two most commonly used models in meta-analysis, though some less frequently used methods, such as Bayesian meta-analysis, and p-value combining approaches, are also available in the literature. In practice, many researchers conduct meta-analysis as follows. First, they perform the Cochran’s test to check the assumption of homogeneity of effects. If the assumption is not rejected, the FE model is used and the results from FE are reported. On the other hand, if this test provides evidence against the homogeneity assumption, the RE model is then used, and the results from the RE model are used. Sometimes, results from both FE and RE models were reported^1^.

An important, but usually unanswered, question in meta-analysis is: how the models used fit the data. Just like in any statistical modeling, the goodness-of-fit test is a critical step to check the model adequacy. The reason we should not ignore this step is that results from an inadequate model may be misleading. Unfortunately, this issue is rarely, if not at all, discussed in practice. There are several possible explanations. First, although many software packages are available, they do not provide GoF tests. Second, many researchers who conduct meta-analysis have limited statistical background and are not aware of this issue and its consequences. Last but not least, no GoF test has been developed for meta-analysis in the literature, though some GoF tests have been proposed for generalized linear mixed models^2^,^3^,^4^,^5^,^6^.

To fill the gap, in this paper we propose some GoF test approaches for meta-analysis to assess if the data are jointly normally distributed, regardless of the type of the mean effects. Through simulation we show that the proposed tests can control type I error rate. We also conduct some simulations to compare the power performance among the proposed tests. Finally, the proposed tests are applied to two real data sets to demonstrate their usefulness.

## Method

Suppose we have statistics y_i_ (e.g., mean difference, effect size, log odds ratio, etc.) and its variance, v_i_, for study i (i = 1, 2, …, K) of K related but independent studies. We want to combine the information from those K studies using the following random effect model:


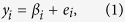


where the independent study-specific effects, *β*_*i*_’s, represent a random sample from a grand normal population with overall mean *μ* (e.g, *μ* = ln(OR) with OR being the overall OR across studies for the binary trait, or the average mean difference across studies for the quantitative trait) and between-study variance *τ*^2^. In other words, we assume 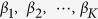 are independently and identically distributed as 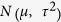. We also assume the study error terms are independent and follow a normal distribution, i.e, 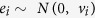. Note that here the variance 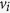 for each individual study may not be the same.

When *τ*^2^ = 0, the random effect model (1) becomes a fixed effect model. Therefore, fixed effect model is a special case of the random effect model. The parameters 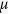 and 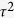 in (1) are usually estimated through a two-step approach. In the first step, 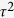 will be estimated using one of many different estimators^7^,^8^,^9^,^10^,^11^,^12^. In general, those approaches give similar results and none of them performs uniformly the best. In addition, among those estimators, the DerSimonian-Laird (DL) method is most commonly used. Therefore, in this paper the DL estimator is used.

The parameter, *τ*^2^, which measures the between-study variance, is estimated by^10^:


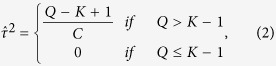


where 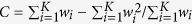, 
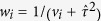
, and 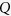 is the statistic of the Cochran’s test for homogeneity^13^,^14^ where the null hypothesis is 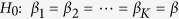. Q is defined as follows:


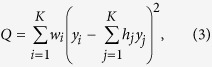


where 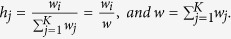


Note that under the normality assumption in model (1) and the null hypothesis 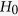 for the Cochran’s test, Q has a chi-square distribution with degrees of freedom (df) equal to K-1. A large value of Q (or a small p-value from the Cochran’s test) indicates the lack of fit of the fixed effect model; a random effect model as (1) is then usually performed.

In the second step, the overall effect of 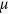 is then estimated by the weighted mean 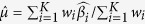, and its variance is estimated by Var
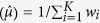
. Then the hypothesis is tested for overall effect across all studies as: *H*_0_: *μ* = 0 *vs H*_1_: *μ* ≠ *0.* The Wald test, 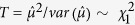 asymptotically under *H*_0_, is the standard test of the hypothesis about the overall mean.

Like any statistical approach, model checking is a critical step as the results from a model with lack of fit may be misleading. Several goodness-of-fit tests have been proposed in the literature for the generalized linear mixed models^2^,^3^,^4^,^5^,^6^, which include the random effect models as special cases. However, unlike the ordinary random effect models where the within cluster error terms are assumed to be independently and identically distributed (iid) as a normal distribution, the RE model (1) in meta-analysis assumes the error terms 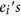 have known and possible different variances. Therefore, those existing goodness-of-fit tests designed for the generalized linear mixed models are inapplicable for the random effect model (1) in meta-analysis.

To fill this gap, we propose some new goodness-of-fit tests for the random effect model (1). It can be easily shown that under model (1), 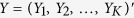 has a multivariate normal distribution, i.e., 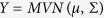, where the covariance matrix 
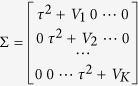
. If the Cochran test statistic Q is large, from (2) we know that 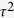 is expected to be large as well. On the other hand, if 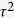 is large enough, the covariance matrix 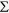 can be approximated by a matrix with all diagonal elements equal to a constant. In this case the y_i_’s can be seen as approximately K iid samples from a normal distribution. Therefore, the normality tests can be used to check the goodness-of-fit for model (1). In this paper, we propose several goodness-of-fit tests for model (1) based on the following popular and powerful normality tests: the Anderson-Darling (AD) test^15^,^16^, the Cramer-von Mises (CvM) test^15^,^17^,^18^, and the Shapiro-Wilk (SW) test^19^. In general, from model (1) the y_i_’s are samples from K independent but not identical normal distributions; the p-values from the normality tests are not accurate. However, to overcome this difficulty we can estimate those p-values based on resampling approaches, such as parametric bootstrap. Specifically, the proposed tests are conducted by the following steps:

**Step 1**. For given 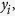 (i = 1, 2, …, K), calculate the statistics ad_0_, cvm_0_, and sw_0_, from the AD, CvM, and SW tests, respectively;

**Step 2.** Estimate 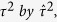 using (2);

**Step 3**. Resample B sub-samples from 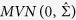, where 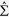 is the estimate of 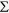 with 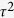 being replaced by its estimate 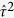. For each sample j (j = 1, 2, …, B), calculate statistics, ad_j_, cvm_j_, and sw_j_ using the AD test, the CvM test, and the SW test, respectively;

**Step 4**. The p-values are estimated by the portions of ad_j_, cvm_j_, and sw_j_, which are greater than ad_0_, cvm_0_, and sw_0_, respectively.

Note that the parameter 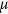 in model (1) has no effect on the normality tests, and therefore, in Step 3 we can simulate data from a multivariate normal distribution with mean vector equal to 0, instead of 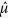.

To assess the performance of the proposed tests, we estimate their type I error rates and the detecting powers using simulated data. We also illustrate the use of the proposed tests through two real data applications.

## Results

### Simulation results

In the simulation study, we assume there are K (K = 5, 10, 15, 20, 30, 50) independent studies. We also assume the variances (v_i_) of the K effects are random samples from the uniform distribution U(*a, b*) with different values of *a* and *b*. To estimate the type I error rate, for the null hypothesis the parameter 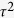 takes three values 0, 0.1, and 1.0. The pair of (*a, b*) take values (0.1, 1.0), (0.01, 0.1), and (0.001, 0.01). Without loss of generality, 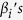 are assumed iid samples from the normal distribution N(0,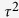). In our simulation study, we use B = 1,000 bootstraps to estimate p-value. The significance level 0.05 and 1,000 replicates are used to estimate the type I error rate and power.

Table 1 reports the estimated type I error rates for each of the three methods. It clearly shows that the proposed tests control type I error rate around the nominal level 0.05 no matter whether the effects are homogeneous (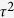 = 0), somehow heterogeneous (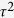 is relatively small, e.g., 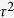 = 0.1 while *a* = 0.1, *b* = 1), or highly heterogeneous (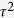 is relatively large, e.g., 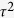 = 0.1 while *a* = 0.001 and *b* = 0.01).

When assessing the detecting power, for the alternative hypothesis, we assume 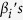 in model (1) have the following distributions: (a) uniform U(-1, 1), (b) log-normal LN(0, 1), (c) double exponential or Laplace DE(0, 1), (d) Cauchy Cauchy(0, 1), and (e) t-distribution with df = 1, T_1_. For the error term, 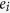, it is independently sampled from a normal distribution with mean 0 and variance v_i_, which is a random sample from the uniform distribution U(*a*, *b*). The values of the pair of (*a*, *b*) are set to be (0.001, 0.01), (0.001, 0.1), (0.001, 1), and (0.001, 10).

Tables 2, 3, 4, 5, 6 report the estimated power values from each method under different settings. It can be seen that when study size (K) is small, all methods have relatively low power values. However, the detecting power increases when the sample size increases. In general, the power decreases when the variances v_i_’s and their heterogeneity increase (e.g, *b* increases). From the simulation results, we can observe that these three methods have similar performance, and none of them is uniformly more powerful than the other two under all conditions.

### Real data application

We use two real data sets of meta-analysis to illustrate the usefulness of the proposed tests. Both of the original data sets gave the estimated odds ratio (OR) and its 95% confidence interval (CI) for each study included in the meta-analyses. Table 7 lists the data set 1 from a meta-analysis of 12 trials that exam the effect of patient rehabilitation designed for geriatric patients on functional outcome improvement. The data were taken from Figure 4 of Riley *et al.*^20^, part of the Figure 2 of Bachmann *et al.*^21^. The p-value from the Cochran’s test for homogeneity was 0.021. Therefore, the authors ran the random effect model and estimated the overall OR as 1.36 with 95% CI (1.08, 1.71)^20^,^21^. It should be pointed out that with small sample size like K = 12 here, the Cochran test is anti-conservative^22^. Table 8 lists the data set 2, which were taken from Figure 2 of Danese and Tan^23^. In data set 2 there are 44 studies investigating the association between childhood maltreatment and obesity. The p-value from the Cochran’s test for homogeneity was very small (<0.0001); the authors then chose random effect model in their meta-analysis. The overall OR was estimated as 1.36 with 95% CI (1.26, 1.74).

In order to use the proposed tests, for data of OR and its 95% CI, as in Tables 7, 8, we first take the logarithm of the OR to get log(OR) and then use log(U/L)/3.92 to get the standard error (square root of v_i_) for each individual study, where U and L are the upper and lower limits of the 95% CI for OR. The above estimates of log(OR) and 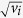 are appropriate as the 95% CI of the odds ratio is usually calculated from 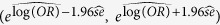, namely, the 95% CI for the log(OR) is 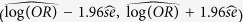, where 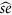 is the estimated standard error of 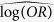. For data set 1, B = 10^5^ bootstraps were used, and the p-values were 0.025, 0.018, and 0.039 from the three proposed goodness-of-fit tests AD, CvM, and SW, respectively. Those small p-values suggest that the random effect model may not be adequate for this data set. For data set 2, the p-values were 0.047, 0.037, and 0.048 from the AD test, the CvM test, and the SW test, respectively. The goodness-of-fit of the random effect model in the meta-analysis was also rejected at significance level 0.05 by all of the three proposed tests.

## Discussion

Meta-analysis is an important and useful tool for combining information from related studies. However, blindly using the tool may result in misleading results. Model checking is a critical step and should not be ignored. The goodness-of-fit tests proposed in this paper provide useful tool to check model adequacy in meta-analysis.

In practice, the Cochran’s test for homogeneity is usually performed in meta-analysis to see whether or not the fixed effect model fits the data. In general, if the hypothesis of homogeneity is rejected, the assumption of the fixed effect model is questionable. As a result, the random effect model is then usually used. However, little has been found in the literature about the adequacy of the random effect model in meta-analysis. Our proposed goodness-of-fit tests fill the gap.

It should be pointed out that the Cochran’s test for homogeneity was not a formally goodness-of-fit test for the fixed effect model, though in many situations rejecting the hypothesis of homogeneity indicates the inadequacy of the fixed effect model. This is because the Cochran’s test assumes individual effects are from normal distributions. When the sample size for each individual study is large, this assumption may be valid. However, when sample sizes are small for some studies, the effects from those studies may not follow the normal distribution. Therefore, it is possible that in a meta-analysis the Cochran’s test does not reject the homogeneity assumption, but the goodness-of-fit tests indicate the lack of fit for both fixed effect and random effect models.

In a meta-analysis, if there is evidence of lack of fit for the random effect model, which includes fixed effect model as a special case, what should we do next? Viechtbauer agued that even if the homogeneity assumption was rejected, it is still valid to use weighted or unweighted mean to estimate the overall effect for the K studies^1^. In addition, many methods based on combining p-values can be used as well in this situation^24^,^25^,^26^,^27^.

Lastly, the proposed tests can be easily extended to check the normality assumption of the random effects in generalized linear mixed models. Furthermore, the proposed tests can also be used to check the random effect assumption in the meta-regression analysis, which is a special case of the generalized linear mixed model and extends the random effect model (1) by adding some fixed effects of study-level covariates.

## Additional Information

**How to cite this article**: Chen, Z. *et al.* Goodness-of-fit test for meta-analysis. *Sci. Rep.* **5**, 16983; doi: 10.1038/srep16983 (2015).

## Figures and Tables

**Table 1:** Estimated type I error rates for each method under settings with different number of studies (K), and parameters (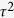, *a, b*) from 1,000 replicates at significant level 0.05.

K	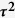 , a, b	AD	CvM	SW	K	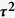 , a, b	AD	CvM	SW
5	0, 0.1, 1	0.052	0.053	0.053	20	0, 0.1, 1	0.051	0.051	0.050
	0.1, 0.1, 1	0.050	0.049	0.049		0.1, 0.1, 1	0.041	0.040	0.039
	1, 0.1, 1	0.041	0.038	0.042		1, 0.1, 1	0.037	0.041	0.037
	1, 0.01, 0.1	0.051	0.048	0.050		1, 0.01, 0.1	0.052	0.052	0.047
	1, 0.001, 0.01	0.048	0.047	0.048		1, 0.001, 0.01	0.043	0.040	0.044
10	0, 0.1, 1	0.044	0.041	0.044	30	0, 0.1, 1	0.046	0.050	0.038
	0.1, 0.1, 1	0.048	0.056	0.043		0.1, 0.1, 1	0.065	0.065	0.063
	1, 0.1, 1	0.054	0.057	0.050		1, 0.1, 1	0.047	0.040	0.050
	1, 0.01, 0.1	0.062	0.058	0.060		1, 0.01, 0.1	0.045	0.048	0.050
	1, 0.001, 0.01	0.051	0.048	0.052		1, 0.001, 0.01	0.056	0.062	0.054
15	0, 0.1, 1	0.038	0.045	0.046	50	0, 0.1, 1	0.037	0.036	0.047
	0.1, 0.1, 1	0.052	0.060	0.045		0.1, 0.1, 1	0.055	0.058	0.058
	1, 0.1, 1	0.049	0.046	0.050		1, 0.1, 1	0.046	0.049	0.046
	1, 0.01, 0.1	0.045	0.048	0.052		1, 0.01, 0.1	0.051	0.050	0.046
	1, 0.001, 0.01	0.051	0.044	0.055		1, 0.001, 0.01	0.063	0.059	0.058

**Table 2:** Estimated power for each method under settings with different number of studies (K), and different values of (a, b) from 1,000 replicates at significant level 0.05.

K	a, b	AD	CvM	SW	K	a, b	AD	CvM	SW
5	0.001,0.01	0.058	0.056	0.065	20	0.001,0.01	0.174	0.155	0.177
	0.001,0.1	0.055	0.052	0.056		0.001,0.1	0.088	0.080	0.079
	0.001,1	0.043	0.040	0.043		0.001,1	0.048	0.034	0.048
	0.001,10	0.047	0.047	0.047		0.001,10	0.047	0.042	0.056
10	0.001,0.01	0.078	0.074	0.084	30	0.001,0.01	0.263	0.202	0.308
	0.001,0.1	0.069	0.064	0.066		0.001,0.1	0.118	0.110	0.117
	0.001,1	0.039	0.042	0.036		0.001,1	0.044	0.045	0.040
	0.001,10	0.050	0.053	0.056		0.001,10	0.039	0.036	0.045
15	0.001,0.01	0.104	0.091	0.114	50	0.001,0.01	0.496	0.374	0.622
	0.001,0.1	0.074	0.069	0.067		0.001,0.1	0.248	0.212	0.241
	0.001,1	0.037	0.044	0.029		0.001,1	0.038	0.038	0.034
	0.001,10	0.058	0.057	0.050		0.001,10	0.046	0.048	0.056

Here 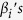 are generated from the uniform distribution U(−1, 1) and the e_i_’s from normal distribution N(0, v_i_).

**Table 3:** Estimated power for each method under settings with different number of studies (K), and different values of (a, b) from 1,000 replicates at significant level 0.05.

K	a, b	AD	CvM	SW	K	a, b	AD	CvM	SW
5	0.001,0.01	0.230	0.222	0.232	20	0.001,0.01	0.893	0.874	0.911
	0.001,0.1	0.231	0.221	0.236		0.001,0.1	0.863	0.827	0.882
	0.001,1	0.150	0.149	0.146		0.001,1	0.612	0.593	0.636
	0.001,10	0.073	0.074	0.074		0.001,10	0.184	0.180	0.187
10	0.001,0.01	0.588	0.566	0.610	30	0.001,0.01	0.985	0.977	0.993
	0.001,0.1	0.521	0.505	0.547		0.001,0.1	0.961	0.953	0.967
	0.001,1	0.339	0.327	0.337		0.001,1	0.800	0.784	0.806
	0.001,10	0.118	0.112	0.125		0.001,10	0.279	0.257	0.289
15	0.001,0.01	0.769	0.750	0.813	50	0.001,0.01	1.000	0.999	1.000
	0.001,0.1	0.728	0.709	0.759		0.001,0.1	0.995	0.994	0.997
	0.001,1	0.517	0.494	0.538		0.001,1	0.942	0.927	0.943
	0.001,10	0.161	0.154	0.170		0.001,10	0.364	0.347	0.385

Here 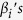 are generated from the log-normal distribution LN(0, 1) and the e_i_’s from normal distribution N(0, v_i_).

**Table 4:** Estimated power for each method under settings with different number of studies (K), and different values of (a, b) from 1,000 replicates at significant level 0.05.

K	a, b	AD	CvM	SW	K	a, b	AD	CvM	SW
5	0.001,0.01	0.073	0.071	0.077	20	0.001,0.01	0.274	0.262	0.270
	0.001,0.1	0.078	0.080	0.074		0.001,0.1	0.256	0.252	0.259
	0.001,1	0.065	0.064	0.068		0.001,1	0.152	0.142	0.151
	0.001,10	0.068	0.066	0.063		0.001,10	0.053	0.051	0.051
10	0.001,0.01	0.173	0.164	0.166	30	0.001,0.01	0.365	0.372	0.353
	0.001,0.1	0.159	0.154	0.157		0.001,0.1	0.332	0.324	0.335
	0.001,1	0.094	0.086	0.096		0.001,1	0.185	0.178	0.212
	0.001,10	0.062	0.057	0.058		0.001,10	0.065	0.070	0.053
15	0.001,0.01	0.215	0.210	0.206	50	0.001,0.01	0.527	0.511	0.509
	0.001,0.1	0.215	0.211	0.212		0.001,0.1	0.519	0.514	0.508
	0.001,1	0.121	0.120	0.123		0.001,1	0.268	0.256	0.292
	0.001,10	0.052	0.049	0.047		0.001,10	0.078	0.078	0.075

Here 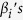 are generated from the double exponential distribution DE(0, 1) and the e_i_’s from normal distribution N(0, v_i_).

**Table 5:** Estimated power for each method under settings with different number of studies (K), and different values of (a, b) from 1,000 replicates at significant level 0.05.

K	a, b	AD	CvM	SW	K	a, b	AD	CvM	SW
5	0.001,0.01	0.289	0.296	0.282	20	0.001,0.01	0.845	0.843	0.834
	0.001,0.1	0.299	0.304	0.295		0.001,0.1	0.878	0.870	0.858
	0.001,1	0.266	0.268	0.252		0.001,1	0.828	0.824	0.826
	0.001,10	0.203	0.205	0.202		0.001,10	0.678	0.660	0.676
10	0.001,0.01	0.572	0.581	0.549	30	0.001,0.01	0.969	0.967	0.963
	0.001,0.1	0.594	0.591	0.565		0.001,0.1	0.962	0.962	0.954
	0.001,1	0.573	0.571	0.550		0.001,1	0.939	0.937	0.938
	0.001,10	0.407	0.405	0.409		0.001,10	0.831	0.822	0.839
15	0.001,0.01	0.789	0.786	0.772	50	0.001,0.01	1.000	1.000	0.999
	0.001,0.1	0.770	0.764	0.755		0.001,0.1	1.000	1.000	0.998
	0.001,1	0.716	0.716	0.707		0.001,1	0.994	0.992	0.994
	0.001,10	0.548	0.541	0.548		0.001,10	0.921	0.911	0.927

Here 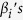 are generated from the double exponential distribution DE(0, 1) and the e_i_’s from normal distribution N(0, v_i_).

**Table 6:** Estimated power for each method under settings with different number of studies (K), and different values of (a, b) from 1,000 replicates at significant level 0.05.

K	a, b	AD	CvM	SW	K	a, b	AD	CvM	SW
5	0.001,0.01	0.282	0.284	0.273	20	0.001,0.01	0.879	0.873	0.868
	0.001,0.1	0.277	0.288	0.275		0.001,0.1	0.875	0.865	0.863
	0.001,1	0.273	0.282	0.261		0.001,1	0.829	0.817	0.810
	0.001,10	0.219	0.220	0.211		0.001,10	0.632	0.615	0.641
10	0.001,0.01	0.620	0.621	0.600	30	0.001,0.01	0.965	0.963	0.953
	0.001,0.1	0.608	0.607	0.591		0.001,0.1	0.961	0.961	0.957
	0.001,1	0.539	0.535	0.531		0.001,1	0.947	0.942	0.938
	0.001,10	0.388	0.383	0.387		0.001,10	0.816	0.799	0.823
15	0.001,0.01	0.806	0.802	0.779	50	0.001,0.01	0.998	0.998	0.996
	0.001,0.1	0.783	0.789	0.758		0.001,0.1	0.996	0.996	0.997
	0.001,1	0.725	0.721	0.718		0.001,1	0.993	0.991	0.994
	0.001,10	0.549	0.540	0.542		0.001,10	0.933	0.921	0.932

Here 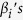 are generated from the T_1_ distribution and the e_i_’s from normal distribution N(0, v_i_).

**Table 7:** Data set 1.

Study	OR	95% CI	study	OR	95% CI	study	OR	95% CI
1	1.11	0.51,2.39	5	0.88	0.39,1.95	9	1.06	0.63,1.79
2	0.97	0.78,1.21	6	1.28	0.71,2.30	10	2.95	1.54,5.63
3	1.13	0.73,1.72	7	1.19	0.69,2.08	11	2.36	1.18,4.72
4	1.08	0.42,2.75	8	3.82	1.37,10.60	12	1.68	1.05,2.70

Estimated odds ratio and its 95% CI from each study. Data were taken from Bachmann *et al.* and Riley *et al.*^20^,^21^.

**Table 8:** Data set 2.

Study	OR	95% CI	study	OR	95% CI	study	OR	95% CI
1	3.16	1.69,5.94	16	1.90	1.10,3.40	31	1.11	0.66,1.87
2	3.91	2.16,7.13	17	1.26	1.13,1.40	32	0.67	0.35,1.28
3	1.42	1.03,1.97	18	1.66	1.04,2.62	33	1.50	1.10,1.90
4	2.51	1.17,5.53	19	1.03	0.63,1.69	34	1.31	0.92,1.87
5	2.40	1.16,3.60	20	2.85	1.06,4.64	35	1.20	0.87,1.65
6	9.80	3.50,28.20	21	0.94	0.72,1.24	36	1.60	1.05,2.43
7	1.20	0.70,2.05	22	2.05	1.59,2.63	37	1.31	1.25,1.38
8	4.66	1.65,13.16	23	1.33	1.11,1.60	38	1.58	1.10,2.27
9	2.25	0.98,5.21	24	1.16	0.88,1.52	39	1.99	1.11,3.55
10	1.39	1.19,1.62	25	1.18	0.92,1.51	40	1.78	1.05,3.04
11	1.34	1.03,1.75	26	1.56	1.03,2.36	41	1.09	1.05,1.14
12	0.91	0.55,1.49	27	1.02	0.77,1.37	42	1.13	0.55,2.37
13	1.08	0.95,1.22	28	0.96	0.74,1.25	43	0.71	0.45,1.10
14	0.86	0.46,1.62	29	1.41	1.00,2.00	44	1.29	1.13,1.49
15	2.23	1.16,4.31	30	1.56	0.96,2.59			

Estimated odds ratio and its 95% CI from each study. Data were taken from Danese and Tan^23^.
